# Correlation between Nonlinear Optical Effects and Structural Features of Aurone-Based Methacrylic Polymeric Thin Films

**DOI:** 10.3390/ma15176076

**Published:** 2022-09-01

**Authors:** Karolina Waszkowska, Anastasiia Krupka, Vitaliy Smokal, Oksana Kharchenko, Anna Migalska-Zalas, Mykhaylo Frasinyuk, Robert Wielgosz, Anatoliy Andrushchak, Bouchta Sahraoui

**Affiliations:** 1 Laboratory MOLTECH-Anjou, University of Angers, CNRS UMR 6200, 2 Bd Lavoisier, CEDEX 01, 49045 Angers, France; 2 Faculty of Chemistry, Taras Shevchenko National University of Kyiv, 60 Volodymyrska, 01033 Kiev, Ukraine; 3 Faculty of Science and Technology, Jan Długosz University in Częstochowa, Al. Armii Krajowej 13/15, 42-201 Częstochowa, Poland; 4 V.P. Kukhar Institute of Bioorganic Chemistry and Petrochemistry, National Academy of Sciences of Ukraine, 1 Murmanskaya St., 02094 Kiev, Ukraine; 5 Energia Oze Sp. z o.o., ul. Częstochowska 7, 42-274 Konopiska, Poland; 6 Department of Applied Physics and Nanomaterials Science, Lviv Polytechnic National University, 12 Bandery Street, 79013 Lviv, Ukraine; 7 LPHIA, SFR MATRIX, University of Angers, 49000 Angers, France

**Keywords:** methacrylic polymers, aurone-containing polymers, second harmonic generation, third harmonic generation, nonlinear optical susceptibility, first (β) and second (γ) hyperpolarizabilities

## Abstract

In this study, new photonics architectures and aurone-based methacrylic polymers were designed and synthesized for their optical and nonlinear optical properties. The studied polymeric thin films were deposited by spin coating method. SHG and THG effects were measured via Maker fringe technique in transmission mode and determined using theoretical models. Investigations involved the theoretical quantum chemical calculation of dipole moments, frontier molecular orbital HOMO and LUMO energies, and first (β) and second (γ) hyperpolarizabilities. We determined the impact of the substitution in the para position of the phenyl ring and at the dipole moment of the chromophore on the nonlinear optical properties of the investigated polymers. The presented theoretical and experimental studies provide important information with respect to the design of methacrylic-based polymeric thin film devices and supplement existing knowledge with respect to their nonlinear behaviour.

## 1. Introduction

Modern technical methods are very important in the design of new high-performance materials that will contribute to technological development. Important aspects of technological applications include the synthesis of polymers with desired properties and their response during interaction with light. Moreover, polymeric materials are used as excellent nonlinear optical and optical data storage materials, among other applications [1,2,3,4,5,6,7]. However, among the most interesting synthetic organic compounds are photochromic polymers. Their unique properties include the ability to transform into other forms, in addition to unique spectroscopic properties [8,9]. Furthermore, changes in photochromic polymers can be induced by various photochemical and physical processes through the content of specific chromophore groups that affect changes in dipole moments, the refractive index or structural properties [10,11,12,13]. Moreover, polymers with a conjugated fragment in the chromophore moiety are promising organic materials due to the possibility of relatively easily modifying their structure. The change of substituent in the molecule of the chromophore leads to a change in the absorption spectra. Using these approaches, it is possible to modify resulting polymers for specific applications. In addition, the introduction of an electron-donor or electron-acceptor substituent in the chromophore fragment leads to changes in intermolecular charge transfer, which result in changes not only in linear but also nonlinear properties [14]. Polymeric materials that contain azobenzene or coumarins in their structure are widely studied due to their unique physicochemical properties [15,16,17,18,19,20].

In previous studies have reported the NLO (nonlinear optical) properties of new methacrylic copolymers with photoactive styrylquinoline fragments, benzylidene fragments [21] and azo fragments [22]. The results of these studies confirm the influences of isomeric structure, steric factors and the nature of substituents in conjugated systems, as well as nonlinear optical properties [23]. We have also shown that the significant influences of the electron-withdrawing effect of the NO_2_ group in the styrylquinoline fragment of the copolymer lead to changes in NLO response toward an increase due to more effective charge transfer, which is related to improved electron delocalization. In this respect, new types of side-chain polymers with photochromic aurone moieties with remarkable optical properties were synthesized. The present work is a continuation of our previously published synthesis and spectroscopic studies of aurone-containing polymers [24].

**Figure 1:**
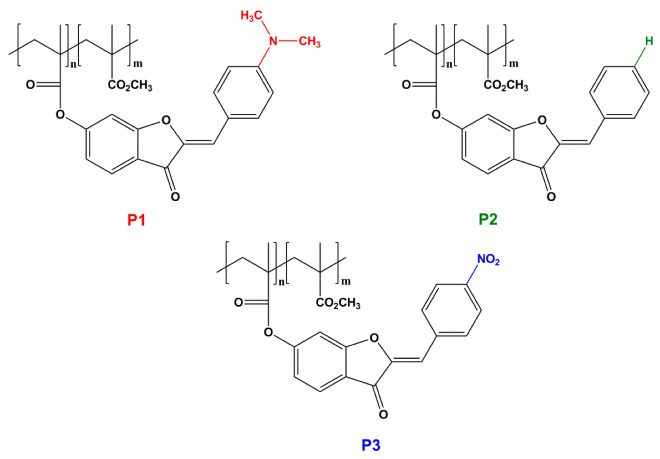
Aurone-based methacrylic polymers.

Herein, we investigated a number of side-chain aurone methacrylic polymers (Figure 1) using the techniques of second harmonic generation (SHG) and third harmonic generation (THG) [25,26,27]. In our previous work [21], we presented, for the first time, the synthesis and characterization of aurone-containing polymers, which are further investigated in this article. Studies of the NLO properties of aurone-containing polymers are of particular interest due to a lack of information on these such properties in these polymers. In the present study, we analyzed and compared results with respect to substituted P1 and P3, as well as aurone moieties and a non-substituted P2 aurone moiety. The significant NLO properties, as well as the optimal composition for the design of new, highly efficient nonlinear optical polymers, were determined. Moreover, the obtained results are expected to provide useful information with respect to the ratio of influences of the chemical structure of the investigated polymers on their nonlinear optical properties, as well as the prospects of using aurone-containing polymers in optical switching applications [28,29,30]. The second important aim of this work was to verify the theoretically obtained results within the framework of density functional theory calculated for the gas phase with experimental measurements and clarify the origin of NLO phenomena in the investigated systems.

## 2. Materials and Methods

### 2.1. Samples Preparation

The general synthetic procedures of 6-hydroxyaurones and 6-methacryloxyaurones are presented elsewhere [24]. The relevant polymers were obtained by free-radical polymerization of previously synthesized monomers. The chemical structures of the corresponding dyes and copolymers were confirmed by ^1^H NMR spectroscopy [24]. The ratio of the comonomer units in the polymers was controlled by the initial ratio of aurone monomers to methyl methacrylate.

Polymeric thin films were obtained on a glass surface by spin coating technique. The glass substrates were conscientiously cleaned with a commercial surfactant using ultrasonication. Solutions of polymers in 1,1,2-trichloroethane 100 g/L were casted on BK7 glass slides. Deposition was carried out on a spin coater (Spin200i, POLOS) at 1000 rpm. Samples of thin films after deposition on the glass were heated at 70 °C for 60 min to remove any remaining solvent. The thickness of the polymeric thin films was measured using a profilometer (Dektak 6M) and recorded as 500 nm, 300 nm and 317 nm for **P1**, **P2** and **P3,** respectively.

### 2.2. SHG and THG Experiments

**Figure 2:**
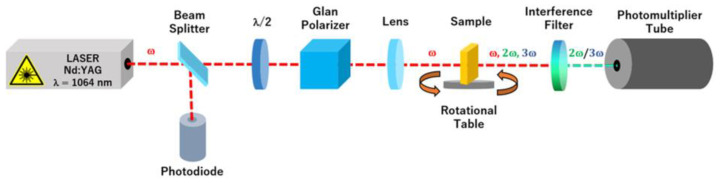
Experimental setup for SHG and THG measurement. After passing the sample, the laser beam was cut off with an interference filter that does not pass radiation at this wavelength. To collect the SHG signal, a 532 nm filter was used, whereas a 355 nm filter was used for the THG signal.

Second and third harmonic generation (SHG and THG, respectively) experiments were carried out by means of the well-known Maker fringe experimental setup in transmission mode with a fundamental laser beam (Figure 2). A picosecond Nd:YAG laser (PL2250, Ekspla, Vilnius, Lithuania) light source was used at wavelength of 1064 nm with a diameter of 0.4 mm. The pulse energy of the laser beam was 100 μJ, with a pulse duration of 30 ps, power density of 3.6 kW/m^3^ and frequency of 10 Hz. The intensities of the fundamental beam were collected as a function of the rotation angle by rotating samples from −75° to +75° for S and P polarization (vertical and horizontal, respectively).

### 2.3. Corona Poling Technique

Second harmonic generation strongly depends on material symmetry. In the case of centrosymmetric materials, SHG experiments can be conducted by creating macroscopic noncentrosymmetry, which is achieved by applying an external electric field via corona poling, which forces the orientation of dipole moments toward the electric field line. Thin films were heated to temperature close to the glass transition temperature (T_g_) of 90 °C for **P2** and 95 °C for **P1** and **P3** in order to increase the mobility of the chromophores, and a high voltage of 6 kV was applied for 40 min. Then, still applying the electric field, the system was cooled down to room temperature. Hence, the orientation of the dipole moments remained petrified for an indefinite time while switching off the corona poling experiment. This orientation time depends on the chemical structure and glass transition temperature of the systems.

### 2.4. Theoretical Calculations

The theoretical energy levels of HOMO and LUMO for P1-P3 were calculated using the GAUSSIAN 09 program package [31]. Becke’s functional B3LYP was used for quantum chemical calculations [32] with a polarized and diffused 6-311++G (d,p) basis. Applying this function provided an opportunity for the determination of dipole moments, geometrical optimization and frontier molecular orbital HOMO and LUMO energies. Based on the absence of any negative vibrational frequency, the minimum energies for the calculated basic states were investigated. The above-mentioned approach was used to compute the frequency-dependent first and second hyperpolarizabilities. The extension of d polarization functions on the C and N atoms and p functions on H atoms or diffuse functions are compelling to obtain precisely evaluate hyperpolarizabilities. The frequency-dependent β(−2ω,ω,ω) and γ(−3ω,ω,ω,ω) simulations at ω = 0.04282 a.u. (λ = 1064 nm) were computed using the GAMESS [33] package within the B3LYP/6-311++G (d,p) basis quantum chemical technique for isolated molecules under vacuum conditions. 

## 3. Results and Discussion

### 3.1. Spectroscopic Studies

**Figure 3:**
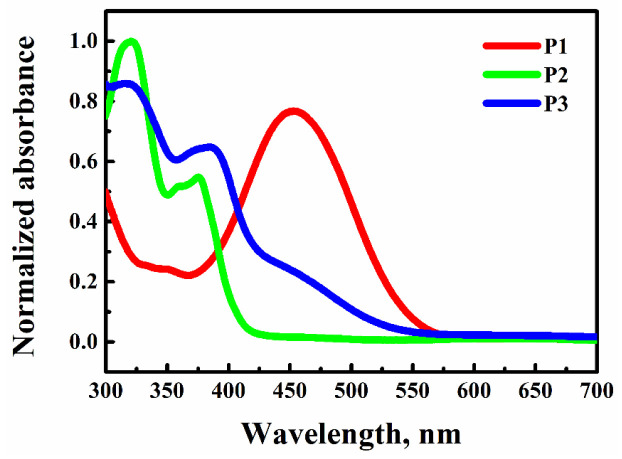
Normalized absorption spectra of **P1**, **P2** and **P3** thin films.

The absorption spectra were recorded on a Shimadzu UV-1800 spectrophotometer in the range of 300–1100 nm. Samples in form of thin films have similar absorption spectra (Figure 3) as samples dissolved in THF [24]. Samples did not absorb at laser wavelengths of 1064 nm and generated a second harmonic at 532 nm, but at 355 nm, which corresponds to THG absorbance, absorbance was not insignificant. This indicates that signals generated during laser irradiation can be reabsorbed by the sample. The calculated absorption coefficients at 355 nm are presented in Table 1.

### 3.2. Nonlinear Optical Studies

**Figure 4:**
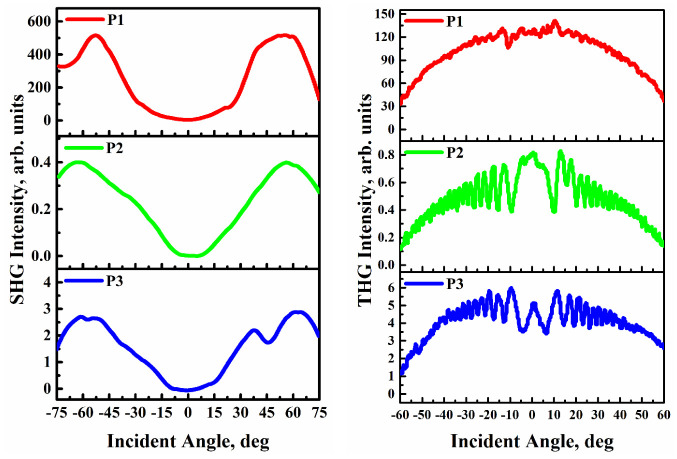
SHG and THG responses as a function of incident angle in **P1**, **P2** and **P3** thin films with P−polarized (SHG) and S−polarized (THG) laser beams.

The intensities of the generated second and third harmonics as a function of the rotation angle are shown in Figure 4. During SHG measurement, Y-cut quartz crystal was used as a reference medium, whereas in the case of THG measurements, silica glass was used as a reference medium.

The second-order nonlinear susceptibilities of the studied thin layers were calculated by comparing the maximum SHG intensities with the maximum intensity of the reference material [34]:

(1) $$\chi^{\left(2\right)}=\chi_{Quartz}^{\left(2\right)}\left(\frac{2}{\pi }\right)\left(\frac{L_{Quartz}^{coh}}{d}\right)\sqrt{\frac{I^{2\omega }}{I_{Quartz}^{2\omega }}}$$

where $I^{2\omega }$ and $I_{Quartz}^{2\omega }$ are the maximum intensity values of the SHG response of the studied material and Y-cut quartz crystal, respectively; $\chi_{Quartz}^{\left(2\right)}$ is the nonlinear optical susceptibility of Y-cut quartz crystal; $L_{Quartz}^{coh}=21\;\mu m$ is the coherent length of Y-cut quartz crystal; d is the thickness of the sample.

Similarly, third-order nonlinear susceptibilities of the studied polymers were determined using the comparative model proposed by Kubodera and Kobayashi [35]. Based on the absorption spectra presented in Figure 3, the equation includes an optical absorption coefficient, expressed as:

(2) $$\chi^{\left(3\right)}=\chi_{Silica}^{\left(3\right)}\left(\frac{2}{\pi }\right)\left(\frac{L_{Silica}^{coh}}{d}\right)\sqrt{\frac{I^{3\omega }}{I_{Silica}^{3\omega }}}\times \left(\frac{\frac{\alpha d}{2}}{1-exp\left(-\frac{\alpha d}{2}\right)}\right)$$

where $\chi_{Silica}^{\left(3\right)}$ is third-order nonlinear susceptibility of the reference material; $L_{Silica}^{coh}=6,7\;\mu m$ is the coherent length of silica glass; *α* is the linear absorption coefficient at 355 nm; $I^{3\omega }$ and $I_{Silica}^{3\omega }$ are the THG maximum-intensity values of aurone-based polymers and silica glass, respectively.

The SHG analysis was performed before and after application of the corona poling technique to the polymeric thin films **P1**–**P3**. We noticed relatively slow disorientation as a function of time as a result of the poling technique; moreover, the response of the investigated systems was meaningful and remained unaffected, even long after the symmetry-breaking technique. This is likely due to the high T_g_ of the investigated polymers. The SHG signal disappeared in the case of samples without corona poling. Nonetheless, we observed enhanced nonlinearities after applying the corona poling technique to all studied polymers. Such results can be attributed to an efficient arrangement of the molecules due to the applied electric field. The approximate measurements were taken via diverse excitation–detection polarization configurations: S and P (Table 2). Because low SHG efficiency was observed for the S-polarization configuration, for all studied polymers, switching from S to P configuration enhanced the nonlinear optical response. This is a consequence of that the fact that the generated second harmonic wave in these materials is completely polarized in the horizontal direction. We found that the values of the second-order NLO susceptibility (*χ*^(2)^) of polymer **P1** were significantly higher in comparison with those of samples **P2** and **P3**, which can be explained by the strong acceptor components in the para position, which have a strong effect on the NLO response. The SHG intensities recorded for aurone-based polymers were compared with the SHG intensities of other polymers with styrilquinoline fragments [23]. The aurone-based **P3** polymer containing an NO_2_ group was compared with the styrilquinoline-based P2 polymer also containing an NO_2_ group. In this case, the SHG response of the aurone-based **P3** polymer is characterized by a higher intensity than that of the styrilquinoline-based polymer. Second-order NLO susceptibility values in the P polarization configuration are more than twice as high: **P3** (aurone) *χ*^(2)^ = 2.91 pm/V and P2 (styrilquinoline) *χ*^(2)^ = 1.25 pm/V [23].

**Figure 5:**
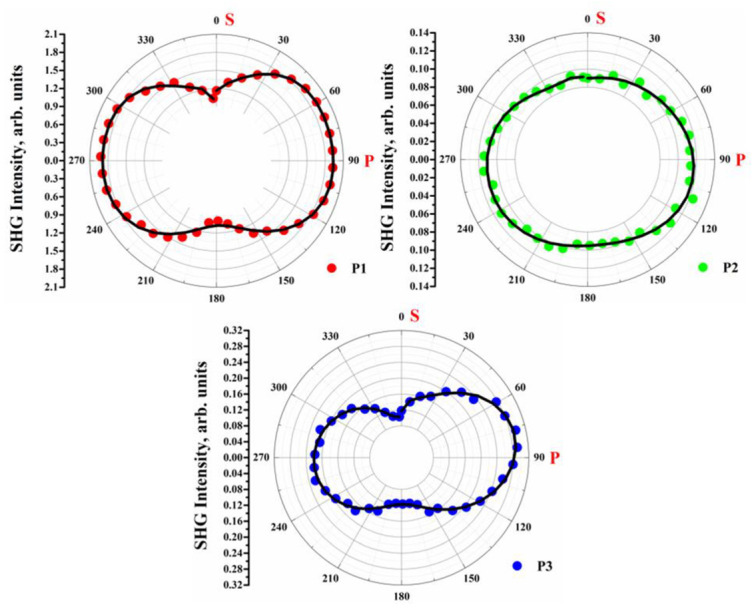
Dependence of generated second harmonic intensities on output laser polarization.

Figure 5 displays the dependence of the signal intensity of the generated second harmonic (in arbitrary units) as a function of the applied laser beam polarization. This was achieved by rotating Glan’s polarizer from 0 to 360 degrees and recording the average SHG intensity during one pulse of the laser beam at an angle corresponding to the maximum intensity of the SHG shown in Figure 4 (around 55°), where 0° and 180° correspond to S polarization, and 90° and 270° correspond to P polarization. Figure 5 shows that for samples **P1** and **P3**, the graph resembles an inverted “8”whereas for sample **P2**, the result forms an ellipse. These results are most consistent with the calculated values of the second-order nonlinear susceptibility, i.e., in the case of thin layers **P1** and **P3,** the *χ*^(2)^ values are much higher for the P polarization than for the S polarization, whereas in the case of sample **P2**, there was no significant discrepancy in the calculated values for P and S polarizations.

In the case of THG, effects the different excitation-detection polarization configurations (S and P) are characterized by weak influences on the third-order NLO susceptibilities of the polymers. In contrast to SHG, the process of generating third harmonics is not dependent on the material’s symmetry. Third-order NLO susceptibilities of polymer thin films **P1** and **P3** are decisively higher than that of polymer **P2** due to the polarity of para-substitutions. For both effects, **P1** polymer has the strongest signal because the nitro substituent (**P3**) is electron-withdrawing, whereas the dimethylamino substituent (**P1**) acts as a donor.

**Figure 6:**
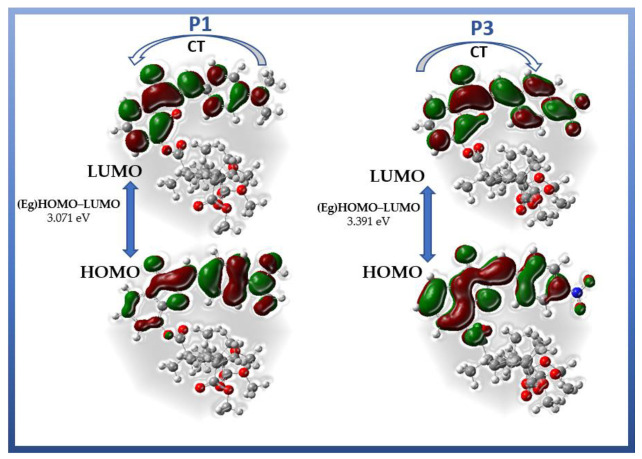
Frontier molecular orbitals HOMO and LUMO of compounds **P1** and **P3** and the intramolecular charge transfer (ICT) process between donor and acceptor groups of compounds **P1** and **P3**.

Figure 6 presents the visualized structures of compounds **P1** and **P3** and represents the intramolecular charge transfer (ICT) of electron density. The properties of dipolar chromophores can easily be tuned by modifying the donor/acceptor groups. The degree of ICT character of an electronic transition significantly differs between chromophores and is affected by substituent groups. The dimethylamino (-N(CH_3_)_2_) group in sample **P1** is an example of a common donor group. Hence, the HOMO for **P1** is mainly located on the benzylidene element, whereas the LUMO is localized on the benzofuran element. The donor dimethylamino substituent displaces electronic clouds towards the polymer part. The nitro group (-NO_2_) in P3 is commonly designed as an electron acceptor in a push–pull system. For the **P3** molecule, the HOMO is located on the benzofuran element, whereas LUMO is located mainly on the benzylidene derivative. The strong electron-acceptor group (NO_2_) influences this electron delocalization. In such a system, efficient intramolecular charge transfer occurs from the donor to the acceptor, leading to the polarization of the molecule. The molecular orbital shape shown in Figure 6 demonstrates that the orbital HOMO of the molecule is a binding π-type orbital, whereas the LUMO is an anti-binding π*-type orbital. Hence, the transition with the highest intensity in the absorption spectrum will be the π-π* character transition. The NLO response is directly related to the extent of the ICT taking place in the D-π-A molecular structure. The electrons forming the π-type bond are delocalized and have significant kinetic energy, which enables easy deformation of the electron cloud of the system. Therefore, the structure of the conjugate molecule provides an electron redistribution pathway between the donor and acceptor. This results in an unbalanced symmetry in the ground state of the electric charge distribution, which is manifested by a significant static dipole moment.

Quantum chemical calculations for the HOMO–LUMO energy gap, dipole moment, HOMO and LUMO energy (see Table 3) provide an opportunity to assess the relationship of nonlinear properties with the structure of the tested materials. It is well-known that nonlinear optical properties are associated with the value of the band gap. The HOMO–LUMO gaps of **P2** and **P3** are relatively higher than energy gap value for **P1**. A high energy gap in **P2** and **P3** suggests that these polymers are less reactive and more stable than molecule **P1**. Lower HOMO–LUMO gaps usually herald better nonlinear properties, which are made visible in these compounds, as measured by the dipole moment.

The dipole moment determining the polarity of molecule **P1** is larger (8.71 D) than that of **P3** (5.98 D) and **P2** (4.71 D). Attaching an appropriate functionality enhances absolute molecular asymmetry in both ground- and excited-state configurations and affects the quadratic and cubic nonlinear optical responses. To identify an interconnection between structural features and nonlinear optical properties, our analysis of the first0 and second-order hyperpolarizabilities was extended. The total first- and second-order hyperpolarizability values were determined using the formula presented in Ref. [39]. The obtained results are presented in Table 4. Usually, an increment in the β value occurs as a result of a bathochromic effect (see Table 1) due to the strong donor ability, which is obvious for polymer **P1**. The obtained theoretical calculations indicate that polymer P1 possess enhanced second-order NLO properties (β_tot_ = 907.044 × 10^−30^ esu). The calculated first-order hyperpolarizability of this polymer compound was compared with the first-order hyperpolarizability of selected organometallic molecules with a similar structure, which are well-known in literature for very high β_tot_ values in terms of 10–1300 × 10^−30^ esu [40,41] and with the first-order hyperpolarizability (β_tot_) of urea sulfamic acid, which is a reference material in second harmonic generation equal to 1.023 × 10^−30^ esu [42]. The obtained values of the first-order hyperpolarizability of the tested molecules are three orders of magnitude higher than that for urea sulfamic acid and comparable with the results obtained for other organometallic molecules.

As shown in Table 4, the value of second-order hyperpolarizability (γ_tot_) of compound **P1** as determined by DFT method (γ_tot_ = 984.868 × 10^−36^ esu) is about six times higher than that of compound **P2** (γ_tot_ = 165.064 × 10^−36^ esu). We compared our calculated hyperpolarizability γ values with those reported in the literature by Leupacher et al. [43] for specific compound methylene blue (γ = 32.00 × 10^−36^ esu) via THG measurements, which revealed that the value of **P1** is thirty times higher than that reported in the literature. High values of the comparable component shows to the delocalization of the electrons in an appropriate direction. The calculated values of β_tot_ and γ_tot_ are in agreement with experimental data reported in the available in literature (see Table 4), considering that the hyperpolarizabilities were calculated without including rotational and vibrational contributions. These studies show that a minor change in the material design can significantly influence the system’s linear and nonlinear optical properties.

## 4. Conclusions

In summary, in this research, we investigated the nonlinear optical properties of new photonic architectures, i.e., aurone-based methacrylic polymers. The second- and third-order nonlinear optical properties were diagnosed using second and third harmonic generation methods. We observed and demonstrated that these interesting photonic architectures, P1, P2 and P3 polymers, have strong nonlinear optical properties. In this paper, we also reported the first- and second-order hyperpolarizabilities calculated within the framework of density functional theory. Calculations performed according to the DFT method accurately reflect the trend of changes in the values obtained using experimental methods and suggest that hyperpolarizability is controlled by donor–acceptor strengths. The calculated and measured values of the NLO properties for the studied compounds are in the order P1 > P3 > P2. Such an arrangement is consistent with enhancement of the γ_tot_ caused by the push–pull effect. In this study, we demonstrated that the donor substituent considerably enhances the SHG and THG effects. Therefore, we can conclude the nonlinear optical response can be controlled relative to molecular structures, and we showed that compound P1, an aurone-based polymer, is a promising photonic architecture for optical switching applications in nonlinear optical devices.

## Figures and Tables

**Table 1:** Absorption peaks and values of absorption coefficients of **P1**, **P2** and **P3** thin films.

Sample	λ_abs_ (nm)	α_(355nm)_ (10^3^ cm^−1^)
P1	452	18.30
P2	320; 375	11.84
P3	316; 382	15.14

**Table 2:** Second- and third-order NLO susceptibilities (*χ*^(2)^, *χ*^(3)^) of **P1**, **P2** and **P3** polymers.

Sample	Thickness d (μm)	*χ*^(2)^, pmV^−1^		*χ*^(3)^, 10^−22^ m^2^V^−2^	
		S	P	S	P
Y-cut quartz [36]	1000	1.00		-	
Silica glass [37]	1000	-		2.00	
P1	0.500	2.03	29.54	229.2	226.1
P2 [38]	0.300	0.34	0.67	3.9	4.2
P3	0.317	0.64	2.91	96.7	97.7

**Table 3:** Dipole moment, HOMO and LUMO energy levels and theoretical bandgap ((E_g_)_HOMO–LUMO_).

Sample	Dipole Moment (D)	HOMO (eV)	LUMO (eV)	(Eg)_HOMO–LUMO_ (eV)
P1	8.71	−5.447	−2.376	3.071
P2	4.71	−6.368	−2.769	3.599
P3	5.98	−6.831	−3.441	3.391

**Table 4:** Frequency-dependent β_tot_(−2ω;ω,ω) and γ_tot_(−3ω;ω,ω,ω) values at ω= 0.042827, a.u. = 1064 nm, B3LYP/6-311++G(d,p) for **P1**–**P3** and comparison with experimental data: *χ*^(2)^ and *χ*^(3)^ at P polarization.

Sample	Calculations B3LYP/6-311++G (d,p)		Experiment	
	β_tot_ × 10^−30^, esu	γ_tot_ × 10^−36^, esu	*χ*^(2)^, pmV^−1^	*χ*^(3)^, 10^−22^ m^2^V^−2^
P1	907.044	984.868	29.54	226.1
P2	114.946	165.064	0.67	4.2
P3	177.302	425.405	2.91	97.7

## Data Availability

The data presented in this study are available on request from the corresponding author.
